# Comparing AI and Manual Segmentation of Prostate MRI: Towards AI-Driven 3D-Model-Guided Prostatectomy

**DOI:** 10.3390/diagnostics15091141

**Published:** 2025-04-30

**Authors:** Thierry N. Boellaard, Roy van Erck, Sophia H. van der Graaf, Lisanne de Boer, Henk G. van der Poel, Laura S. Mertens, Pim J. van Leeuwen, Behdad Dashtbozorg

**Affiliations:** 1 Department of Radiology, Netherlands Cancer Institute, Plesmanlaan 121, 1066 CX Amsterdam, The Netherlands; 2 Technical Medicine, Faculty of Mechanical, Maritime, and Materials Engineering, Delft University of Technology, Mekelweg 2, 2628 CD Delft, The Netherlands; 3 Department of Urology, Netherlands Cancer Institute, Plesmanlaan 121, 1066 CX Amsterdam, The Netherlands; 4 Image-Guided Surgery, Department of Surgery, Netherlands Cancer Institute, Plesmanlaan 121, 1066 CX Amsterdam, The Netherlands; 5 Department of Urology, Amsterdam University Medical Centers, De Boelelaan 1117, 1081 HV Amsterdam, The Netherlands

**Keywords:** prostatic neoplasms, magnetic resonance imaging (MRI), prostatectomy, three-dimensional images, artificial intelligence, tumor segmentation, prostate segmentation

## Abstract

**Background**: Robot-assisted radical prostatectomy (RARP) is a common treatment option for prostate cancer. A 3D model for surgical guidance can improve surgical outcomes. Manual expert radiologist segmentation of the prostate and tumor in prostate MRI to create 3D models is labor-intensive and prone to inter-observer variability, highlighting the need for automated segmentation methods. **Methods**: This study evaluates the performance of the prostate and tumor segmentation using a commercially available AI tool without (fully automated) and with manual adjustment (AI-assisted) compared to manual segmentations on 120 patients, using several metrics, including Dice Coefficient and Hausdorff distance. Tumor detection rates were assessed with recall and precision. **Results**: For the prostate, both the fully automated AI model and AI-assisted model achieved a mean Dice score of 0.88, while AI-assisted had a lower Hausdorff distance (7.22 mm) compared to the fully automated (7.40 mm). For tumor segmentations, the Dice scores were 0.53 and 0.62, with Hausdorff distances of 9.53 mm and 6.62 mm obtained for fully automated AI and AI-assisted methods, respectively. The fully automated AI method had a recall of 0.74 and a precision of 0.76 in tumor detection, while the AI-assisted method achieved 0.95 recall and 0.94 precision. Fully automated segmentation required less than 1 min, while adjustments for the AI-assisted segmentation took an additional 81 s, and manual segmentation took approximately 15–30 min. **Conclusions**: The fully automated AI model shows promising results, offering high tumor detection rates and acceptable segmentation metrics. The AI-assisted strategy improved the relevant metrics with minimal additional time investment. Therefore, the AI-assisted segmentation method is promising for allowing 3D-model-guided surgery for all patients undergoing RARP.

## 1. Introduction

Prostate cancer is the most common cancer among men in Europe, and robot-assisted radical prostatectomy (RARP) is one of the common treatment options [1,2]. Successful radical prostatectomy is defined as a trifecta: the complete removal of the tumor while preserving continence and erectile function. Virtual or printed 3D models derived from prostate MRI have shown several useful applications for planning and guiding RARP in order to achieve this trifecta [3]. For example, 3D models can help with nerve-sparing planning to preserve continence and erectile function [4], guide the location of frozen sections during surgery to allow for secondary resections with negative surgical margins [5], and significantly reduce the incidence of positive surgical margins [6]. However, manual segmentations of the prostate and tumor tissue from the prostate MRI that are necessary for the generation of these 3D models are labor-intensive [7,8]. This highlights the need for automated segmentation methods. AI models have shown good results in prostate cancer detection, usually retrospectively [9]. One study showed superior results compared with 62 reads, although not superior to expert clinical practice reads [10]. These AI algorithms can generate the segmentations of the prostate and tumor needed to create 3D models. Although many AI algorithms have been developed and studied, only a few have become commercially available. One of these commercially available tools is the Prostate MR application on syngo.via (Siemens Healthineers, Forchheim, Germany), which can generate segmentations of both the tumor and prostate. Previous studies have primarily evaluated the diagnostic accuracy of AI algorithms for prostate lesions detection [11,12,13,14,15,16,17]. However, these studies did not evaluate the quality of the segmentation, for example, expressed by factors such as volume, overlap, and centre, factors that are essential for 3D-model-guided surgery. In this study, we compare the relevant voxel-based, lesion-based and volume-based metrics of the fully automated AI segmentations without and with adjustments compared with expert radiologist segmentations as a step towards AI-driven 3D-model-guided RARP.

## 2. Materials and Methods

### 2.1. Study Design

A retrospective cohort design was utilized to evaluate the performance of the Siemens AI tool for prostate and tumor segmentation compared to a manual segmentation performed by an expert radiologist. The study cohort consisted of 142 patients diagnosed with biopsy-proven localized prostate cancer, who were visiting the Netherlands Cancer Institute-Antoni van Leeuwenhoek Hospital (NKI-AVL) from February 2021 to October 2023. All included patients were scheduled for an RARP and had undergone a preoperative prostate MRI that showed at least one lesion with a Prostate Imaging Reporting and Data System (PI-RADS) score of 3–5. Patients were excluded if their MRI was older than six months prior to the RARP or if no nerve sparing was performed. The study was approved by the Institutional Review Board of the hospital (IRBd25-011) and adhered to the Declaration of Helsinki. Informed consent was obtained from all patients.

The MRI scans included in this study were acquired with various MRI machines from different manufacturers, including Philips Medical Systems, Siemens Healthineers, and GE Medical Systems; for details, see Appendix A, Table A1. Our center is part of a large prostate cancer network, ‘Prostate Cancer Network the Netherlands’, consisting of 13 centers with centralized robot-assisted radical prostatectomy performed only in our center. Also, we are a tertiary referral cancer center where second opinions are performed with MRI scans from the referring centers. Therefore, many different MRI machines were involved. All patients underwent T2-weighted MRI, apparent diffusion coefficient (ADC), and diffusion-weighted imaging (DWI) scans. For some of the patients, a dynamic contrast-enhanced (DCE) sequence was employed.

### 2.2. Methodology

#### 2.2.1. Prostate and Tumor Segmentation Process

##### Manual Segmentation

The manual segmentations of the prostate and tumor were performed independently by a certified expert radiologist with over nine years of experience in reading prostate MRI scans. For the manual segmentation, the radiologist used the Vue PACS software v.12, Philips Medical Systems, Best, the Netherlands, Philips. All segmentations were performed on the axial T2 sequence. For tumor detection and size estimation, the T2, DWI, and ADC sequences were used (and DCE if available). In cases where an anomaly was detected on one sequence, the others were reviewed to confirm the lesion’s presence and characteristics. Additional information such as biopsy results (usually) or PSMA PET scans (in some of the patients) was also available. The radiologist used this information to enhance the accuracy of the segmentation masks. In this study, the results of the manual segmentation served as a reference standard (ground truth) for evaluating the performance of the method with the fully automated AI and the AI-assisted method.

##### Fully Automated AI Segmentation

MRI scans were annotated using the Prostate MR reading workflow on syngo.via VB60 (Siemens Healthineers, Erlangen, Germany), which offers an AI-based fully automatic segmentation of the tumor and prostate [11,12,13,14,15,16,17]. To create these segmentations, the MRI scans were loaded into the software and automatically pre-processed using the MR prostate module. Only the axial T2 and the DWI images were used as input for the algorithm. Subsequently, all segmentations were accepted and exported via the biopsy support in an RT Struct format.

##### AI-Assisted Segmentation

After the radiologist had completed the manual segmentation and the AI tool had generated the automated segmentations, the radiologist adjusted the automated segmentations to create the AI-assisted segmentation. In this set of segmentations, the radiologist performed a thorough review and adjustment of the results obtained by the automated AI method. False positives identified by the automated AI method were removed after careful consideration. Conversely, any lesions that the radiologist identified as missed by the automated AI method were manually added to ensure all tumors were captured. For lesions confirmed to be true positives, the radiologist carefully examined the boundaries and made adjustments when deemed necessary. This process was not limited to basic edits; it also involved integrating expert knowledge and cross-referencing with other imaging modalities, such as ADC, DWI, and T2-weighted sequences, to verify the presence and characteristics of the lesions. Furthermore, the time required for these adjustments was systematically measured and recorded to evaluate the efficiency of the AI-assisted approach compared to fully manual segmentation.

#### 2.2.2. Performance Evaluation

Both the fully automatic and the AI-assisted segmentations were evaluated against the reference standard of manual segmentations (ground truth) using various metrics to ensure a comprehensive performance assessment. The metrics included lesion-based metrics (lesion-based recall and precision), voxel-based (Dice score, Hausdorff distance, Average Surface Distance, voxel-based recall and precision), and volume-based (Absolute Volume Difference, Relative Volume Difference).

For all detected and annotated tumor lesions, recall and precision values were calculated to evaluate the model’s performance. Recall represents the proportion of actual positive lesions correctly identified by the model, while precision measures the proportion of positive predictions that were accurate. These metrics provide insight into the model’s ability to detect and correctly classify lesions.

**Figure 1:**
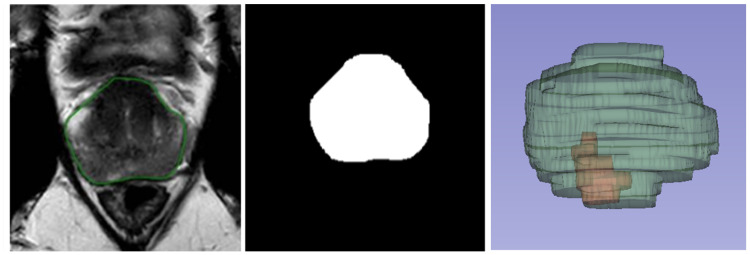
From **left** to **right**: Boundary segmentation on T2-weighted MRI and segmentation mask, and 3D representation of prostate (in green) and tumor (in red).

In addition to the lesion-based metrics, the segmentation performance was also assessed with the voxel-based and volume-based performance metrics. This involved calculating metrics for the prostate and all correctly detected tumor lesions classified as true positives. For each overlapping true positive lesion, a comprehensive set of metrics was applied, including the Dice Coefficient, Hausdorff distance, Average Surface Distance, Euclidean Distance of Centers, and volume-based calculations. These metrics quantified the spatial accuracy, boundary alignment, and volumetric consistency of the segmentations, enabling a detailed evaluation of the AI and AI-assisted approaches relative to the manual reference standard. This multi-level analysis, lesion-based, voxel-based, and volume-based, provided a robust framework for assessing the segmentation performance across varying levels of granularity, ensuring both clinical and technical relevance. A detailed description of all used evaluation metrics including the calculation formulas is included in Appendix A.2. For voxel-wise metric calculations, the manual boundary segmentations were converted into segmentation masks (Figure 1). In these masks, each voxel was assigned a value of 1 if it was classified as part of the segmented prostate or tumor and a value of 0 if it was categorized as background. This binary representation enabled a precise and standardized comparison across segmentation methods.

#### 2.2.3. Data Analysis

Voxel-based and volume-based metrics were calculated only for lesions detected by the fully automated AI or AI-assisted models that overlapped with a lesion identified by the radiologist.

When multiple lesions detected by the automated AI or AI-assisted model overlapped with a single lesion in the manual segmentation, the union of these overlapping lesions was used for metric calculations. Similarly, if multiple manually annotated lesions overlapped with a single automated AI or AI-assisted lesion, the union of the overlapping manual segmentations was considered.

In some instances, the radiologist segmented a single lesion into two separate parts, resulting in distinct segmentations. For lesions appearing very close to one another, the radiologist reviewed the segmentations to identify unintentional separations. If the separation was deemed unintentional, the two parts were combined and treated as a single lesion for analysis.

#### 2.2.4. Statistical Tests

Descriptive statistics were employed to provide a comprehensive evaluation of the model’s performance across multiple metrics. For each metric—Dice Coefficient, Hausdorff distance, Average Surface Distance, recall, precision, Euclidean Distance of Centers, Volume Difference, and Relative Volume Difference—the mean, standard deviation, and median were calculated across all subjects (patients) to capture the overall performance trends.

The Shapiro–Wilk test was applied to assess the normality of data distributions. Based on the results, appropriate statistical tests were selected: either the Wilcoxon signed-rank test or the paired *t*-test was used for paired prostate datasets, while the unpaired *t*-test or the Mann–Whitney U test was employed for comparisons in unpaired lesion datasets, depending on the normality of the data.

To account for multiple comparisons across the eight metrics and control the false discovery rate, *p*-values were adjusted using the Benjamini–Yekutieli procedure. This ensured a rigorous and statistically robust analysis of the model’s performance [18].

#### 2.2.5. Clinical Subgroups

##### PI-RADS

To evaluate the system’s performance across varying clinical contexts, the patient cohort was stratified into clinical subgroups. Each lesion identified by the radiologist was assigned a PI-RADS score ranging from 1 to 5, where a score of 1 represents the lowest likelihood of clinically significant malignancy, and a score of 5 represents the highest likelihood [19]. Higher PI-RADS scores are associated with more visually prominent lesions on MRI scans, potentially making them easier for the AI tool to detect. Based on these scores, the cohort was divided into three subgroups: PI-RADS 3, 4, and 5. For each subgroup, lesion-based recall values were calculated to assess the system’s detection performance. Additionally, for all true positive lesions within each subgroup, the mean, standard deviation, and median were calculated for voxel-based and volume-based performance metrics.

Due to the small sample size in the PI-RADS 3 subgroup (*n* = 5 lesions), this group was excluded from further statistical analysis to avoid unreliable results. For the PI-RADS 4 and 5 subgroups, the normality of metric distributions was assessed using the Shapiro–Wilk test. Depending on the results, either the unpaired *t*-test or the Mann–Whitney U test was applied. To account for multiple comparisons and control the false discovery rate, *p*-values were adjusted using the Benjamini–Yekutieli procedure.

##### Prostate Zone

Given that prostate cancer predominantly arises in the peripheral zone (PZ) [20], the performance of the AI tool was also evaluated across different prostatic zones. Lesions were categorized into the PZ and transition zone (TZ). Only these two zones contained a sufficient number of lesions for meaningful analysis. Recall values were calculated for both zones to assess detection rates. As in the PI-RADS analysis, the Benjamini–Yekutieli procedure was used to adjust for multiple comparisons to correct for the false discovery rate.

## 3. Results

A total of 142 patients were initially included in this study. Of these, all three segmentation methods (manual, fully automated, and AI-assisted) were available for 120 patients. For 22 patients, only the manual segmentation was available because the AI model was unable to annotate the prostate and lesion(s). Errors were caused by the following: split B series (n=16), no low b values (n=2), representation state series present (n=2), wrong T2 series chosen (n=1), and unknown (n=1). This resulted in a primary analysis of 120 patients for whom complete data were available.

### 3.1. Tumor Detection

**Figure 2:**
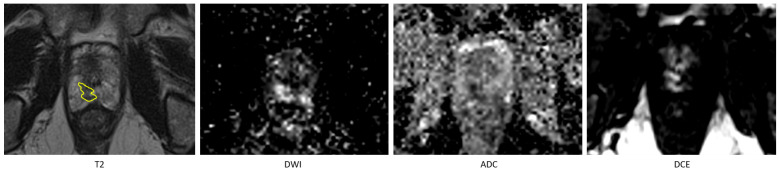
An example of missed lesion (false negative) by the fully automated AI method, where lesion is annotated by the radiologist (in yellow) on T2 hypointense area in the peripheral zone in the right apex with corresponding high signal on the high-B-value DWI and low signal on the ADC and on the DCE early enhancement.

In the comparison between the radiologist and the fully automated AI method, a total of 175 lesions were manually annotated across the 120 included patients. The fully automated AI method achieved a recall of 0.74 and a precision of 0.76 in detecting these lesions. When comparing the radiologist’s segmentations to those of the AI-assisted model, the AI-assisted method demonstrated substantial improved performance, achieving a recall of 0.95 and a precision of 0.94. Detailed metrics are provided in Table 1. An example of missed lesion (false negative) by the fully automated AI method is demonstrated in Figure 2.

### 3.2. Prostate and Tumor Segmentation

Among the voxel-based and volume-based metrics tested, only voxel-based recall in the radiologist-versus-automated AI tumor comparison and Relative Volume Difference in the radiologist-versus-AI-assisted prostate comparison exhibited normal distributions, with *p*-values of 0.13 and 0.12, respectively. All other metrics had *p*-values less than 0.05, indicating non-normal distributions.

**Figure 3:**
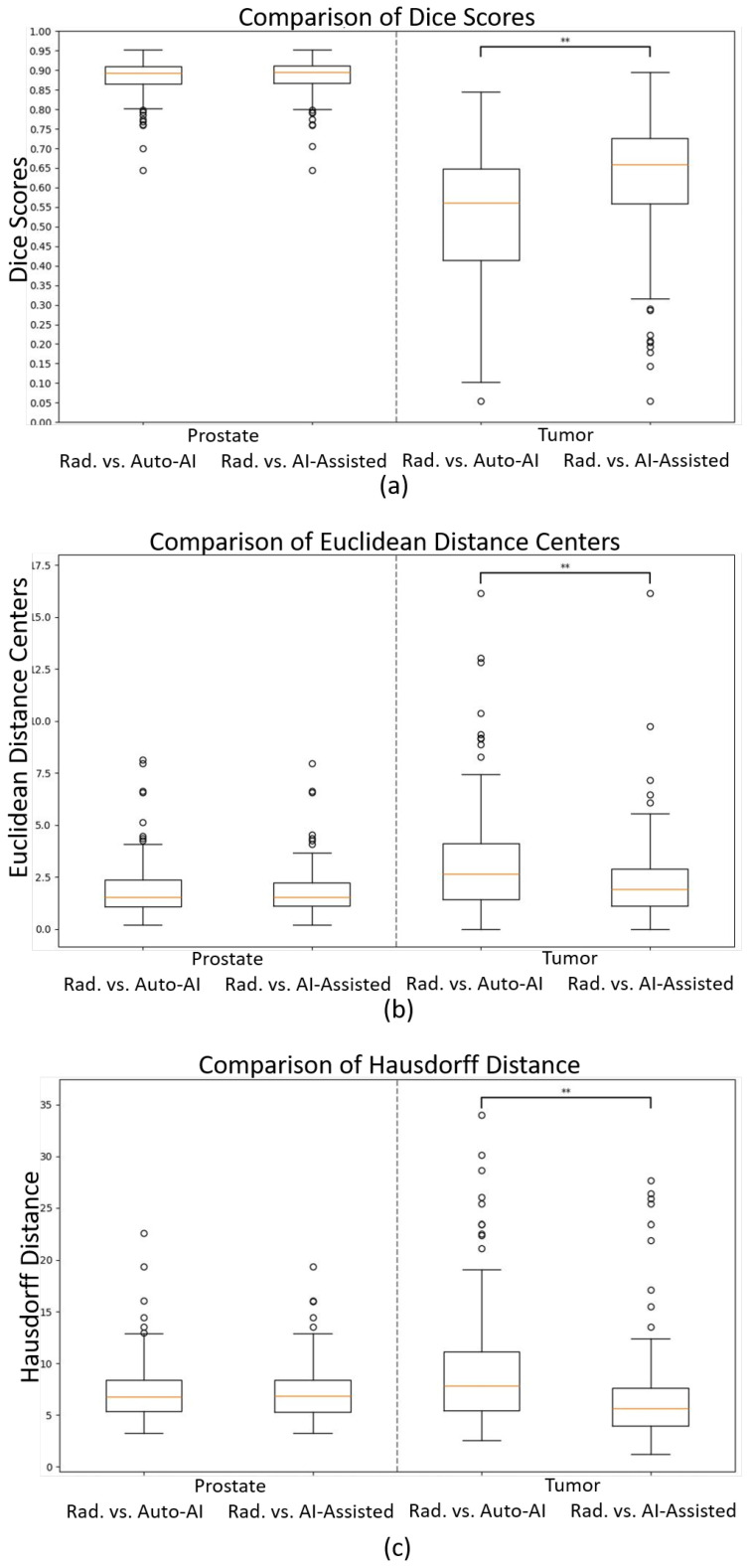
Boxplots of (**a**) Dice scores, (**b**) Euclidean distances of the centers, and (**c**) Hausdorff distances comparing the radiologist (“Rad.”) vs. fully automated AI (“Auto-AI”) and AI-assisted models for prostate (left side) and tumor segmentations (right side). ** p<0.01.

**Figure 4:**
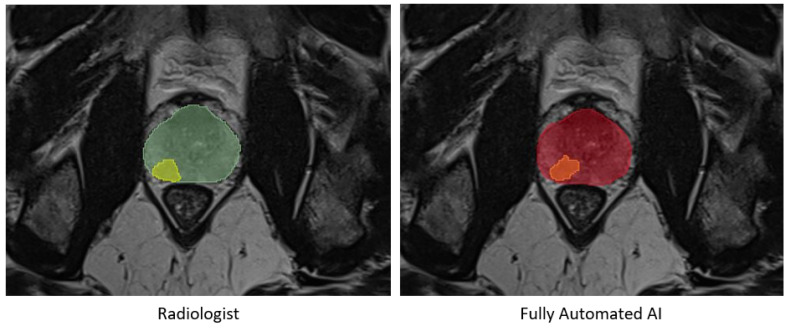
An example of prostate segmentation and tumor segmentation by a radiologist on the **left**, compared to fully automated AI method output on the **right**. Green: Prostate segmentation by radiologist, Yellow: Tumor segmentation by radiologist, Red: Prostate segmentation by fully automated AI, Orange: Tumor segmentation by fully automated AI.

No statistically significant differences were observed in the prostate segmentation comparisons. However, in the tumor comparisons, the statistical tests on all metrics, except for precision, exhibited differences with a *p*-value less than 0.01 between the automated AI and AI-assisted models. Detailed results are presented in Figure 3 and Table 2 and Table 3. An example of prostate and tumor segmentation performed by a radiologist, compared to the fully automated AI method, is shown in Figure 4.

The mean time required for reviewing and adjusting the segmentations was 81 s, with a standard deviation of 65 s and a median of 69 s.

### 3.3. Clinical Subgroups

Of the 175 manually annotated lesions, 34 lesions were excluded from the subgroup analysis due to the unavailability of PI-RADS scores. An additional 31 lesions were excluded because the region of the lesion was not specified. Seven lesions were further excluded because they spanned multiple regions, making them unsuitable for per-region comparisons. Only one lesion was located in the anterior fibromuscular stroma (AFS); this lesion was excluded due to insufficient representation for statistical analysis.

The recall for tumor detection demonstrated an upward trend with increasing PI-RADS scores, indicating improved detection accuracy for lesions with higher malignancy likelihood. Furthermore, recall was notably higher for lesions located in the PZ compared to those in the TZ, suggesting that the system performed better in regions where prostate cancer is more commonly observed. Detailed results are provided in Table 4 and Table 5, highlighting the variations in detection performance across PI-RADS scores and prostate regions. These findings underscore the importance of lesion characteristics and anatomical context in evaluating AI-based segmentation performance.

The performance comparison across different subgroups for lesion segmentation is provided in Appendix A.3.

## 4. Discussion

In this study, we compare the relevant metrics of the AI-based segmentations from commercially available software without and with adjustments of an expert radiologist with manual segmentations of the radiologist as a step towards AI-driven 3D-model-guided RARP.

The prostate segmentation performance of this fully automated model was good with a Dice score of 0.88 and a Hausdorff distance of 7.4 mm. Adjustments to the AI segmentation were only rarely made by the radiologist. In a systematic review of deep learning models for prostate MRI segmentation, reported mean Dice results of 0.90 are very similar to those achieved by the AI software utilized in the present work [21]. Also, similar interobserver differences are found between radiologists (Dice 0.86 and 0.92) [22,23].

For the fully automated lesion segmentation, the metrics of the automated AI method were inferior to those of the prostate volume with a Dice score of 0.53 and a Hausdorff distance of 9.46 mm, reflecting the complexity of this task. Other automated lesion segmentation models have shown comparable results as well, for example, 0.53–0.63 [24,25,26,27], but these were internal validations which generally give better results than external validations such as ours. The lesion segmentation metrics are also similar to the interobserver results reported between radiologists (Dice 0.53–0.55 and Hausdorff 11.02–11.60 mm) [24]. The AI-assisted method improved the relevant metrics: the Dice to 0.62 and the Hausdorff distance to 6.62 mm. While the segmentation performance of the AI model appears comparable to that of the radiologist in terms of descriptive statistics, our statistical analysis revealed important differences. Specifically, several metrics for tumor segmentation—including the Dice Coefficient, Hausdorff distance, Average Surface Distance, and recall—showed statistically significant differences (*p* < 0.05) between the fully automated AI and AI-assisted approaches, as presented in Table 3. These findings underscore that visual or numerical similarity in mean performance does not imply equivalence. Therefore, we emphasize that while the AI-based methods demonstrate promising results, they are not statistically identical to expert radiologist performance. This highlights the added value of AI-assisted approaches and the continued importance of expert oversight in clinical applications.

An AI-assisted method might improve the detection rates compared to a radiologist alone. Ten ‘false positive lesions’ convinced the radiologist that a lesion was present, which could be interpreted as false-negative findings by the radiologist. By subtracting these ten cases from the 41 false positives generated by the AI tool, the precision of the AI tool would increase from 0.76 to 0.81.

The fully automated method provides a fast method for generating segmentations, less than a minute. While manual segmentation requires approximately 15–30 min per patient for the radiologist, the AI-assisted approach reduces this time to an average of 81 s (in addition to the automated generation time).

The AI tool relied solely on T2 and DWI MRI images, whereas the radiologist, in certain cases, used additional information, such as biopsy results and PSMA-PET scans. This makes the AI results even more impressive but also highlights the human ability to integrate a broader range of data sources.

The fully automated method results demonstrate higher recall for lesions with higher PI-RADS scores, reflecting their greater visibility and likelihood of malignancy. Similarly, the better segmentation performance in the PZ compared to the TZ aligns with the higher prevalence and distinct characteristics of prostate cancer in the peripheral zone. These findings suggest that the AI model effectively prioritizes clinically significant lesions and regions, which is consistent with the clinical relevance of PI-RADS and prostate zonal anatomy.

This study is not without limitations. The AI segmentations were compared with a single expert radiologist. The results should be confirmed with multiple radiologists. A part of the prostate MRIs were not segmented by the algorithm, and could therefore not be included in the analysis. Most of these errors (16 of 22) can be easily avoided by making sure the B-values of the DWI sequence are not split into separate series by the MRI machine when sent to the PACS. Furthermore, for optimal validation of AI software, 3D MRI models should also be compared with the ‘gold standard’, i.e., 3D pathology of the prostatectomy specimen. While this research offers a comprehensive assessment of the technical performance of the automated AI and AI-assisted models, it does not address the clinical impact of implementing the AI tool. For example, precise tumor segmentation may not be essential for creating a 3D model that is effective for surgical guidance. Annotation of the approximate location of the tumor may be sufficient to support surgical guidance. Additionally, using both biparametric (without DCE) and multiparametric MRI could have resulted in some variability in PI-RADS scores, because in the multiparametric MRI, the contrast enhancement may have caused some upgrades of the PI-RADS category. The retrospective nature of the study is also a limitation. Furthermore, a relatively small number of patients were included, especially considering the multiple variables investigated. Future studies should address these limitations.

Although the segmentations for 3D-model-guided RARP are important, ideally, more information should be provided by AI. For example, the segmentation of seminal vesicles, urethra, and bladder could improve surgical navigation. Furthermore, AI measurement/segmentation of the neurovascular bundle and membranous urethral length could improve estimated personalized incontinence and erectile dysfunction risk. Providing all these parameters could increase the benefit from AI for prostate MRI evaluation.

## 5. Conclusions

Although the fully automated prostate segmentation method is accurate, tumor detection still requires radiologist evaluation to enhance recall and precision and improve relevant performance metrics. Thus, an AI-assisted method presents a promising approach, while substantially reducing segmentation time. The AI-assisted segmentation method seems a feasible approach to facilitate 3D-model-guided RARP for all prostate cancer patients.

## Figures and Tables

**Table 1:** Lesion detection performance of the fully automated AI and AI-assisted models compared to 175 manually annotated lesions.

Metric	Fully Automated AI	AI-Assisted
True Positive lesions	129	166
False Positive lesions	41	10
False Negative lesions	46	9
Recall_lesion_	0.74	0.95
Precision_lesion_	0.76	0.94

**Table 2:** Comparison of segmentation performance metrics for prostate segmentations between the radiologist and the fully automated AI model versus the AI-assisted model, including mean, standard deviation (STD), median, and *p*-values for statistical significance.

Metric	Radiologist vs. Automated AI			Radiologist vs. AI-Assisted			*p*-Value
	Mean	STD	Median	Mean	STD	Median	
Dice Coefficient	0.88	0.05	0.89	0.88	0.05	0.89	0.51
Hausdorff Distance [mm]	7.40	2.99	6.81	7.22	2.76	6.87	0.80
Average Surface Distance [mm]	1.41	0.66	1.27	1.36	0.62	1.22	0.39
Recall_voxel_	0.82	0.07	0.83	0.82	0.07	0.84	0.61
Precision_voxel_	0.95	0.05	0.97	0.94	0.10	0.97	0.47
Euclidean Distance Centres [mm]	1.94	1.41	1.55	1.84	1.25	1.51	0.52
Difference Volumes (cc)	7.75	8.93	6.53	6.72	4.35	6.27	0.35
Relative Difference Volumes	0.14	0.10	0.14	0.13	0.07	0.13	0.43

**Table 3:** Comparison of segmentation performance metrics for tumor segmentations between the radiologist and the fully automated AI model versus the AI-assisted model, including mean, standard deviation (STD), median, and *p*-values for statistical significance.

Metric	Radiologist vs. Automated AI			Radiologist vs. AI-Assisted			*p*-Value
	Mean	STD	Median	Mean	STD	Median	
Dice Coefficient	0.53	0.17	0.56	0.62	0.16	0.66	<0.0001
Hausdorff Distance [mm]	9.53	5.92	7.82	6.62	4.38	5.62	<0.0001
Average Surface Distance [mm]	1.88	1.26	1.68	1.15	0.87	1.04	<0.0001
Recall_voxel_	0.48	0.20	0.51	0.59	0.18	0.60	<0.0001
Precision_voxel_	0.71	0.22	0.74	0.71	0.19	0.73	1.00
Euclidean Distance Centres [mm]	3.23	2.67	2.65	2.24	1.81	1.91	0.001
Absolute Volume Difference (cc)	1.59	4.91	0.56	0.72	1.26	0.32	0.0004
Relative Volume Difference	0.61	1.20	0.38	0.38	0.81	0.26	<0.0001

**Table 4:** Lesion detection performance metrics of the fully automatic approach by PI-RADS score, including the number of true positives, false negatives, total lesions, and recall values for each PI-RADS category.

PI-RADS Score	True Positive (TP)	False Negative (FN)	Recall
PI-RADS 3	5	5	0.50
PI-RADS 4	38	23	0.62
PI-RADS 5	62	8	0.89

**Table 5:** Lesion detection performance metrics of the fully automatic approach by prostatic region, including the number of true positives, false negatives, total lesions, and recall values for lesions located in the peripheral zone (PZ) and transition zone (TZ).

Region	True Positive (TP)	False Negative (FN)	Recall
PZ	90	28	0.76
TZ	11	7	0.61

## Data Availability

The data presented in this study are available on request from the corresponding author. The data are not publicly available.

## Abbreviations

The following abbreviations are used in this manuscript:

3D	Three-dimensional
ADC	Apparent diffusion coefficient
AI	Artificial intelligence
DCE	Dynamic contrast enhanced
DWI	Diffusion-weighted imaging
FN	False negative
FP	False positive
MRI	Magnetic resonance imaging
PI-RADS	Prostate imaging-reporting and data system
PSMA-PET	Prostate-specific membrane antigen positron emission tomography
PZ	Peripheral zone
RARP	Robot-assisted radical prostatectomy
STD	Standard deviation
TZ	Transitional zone

## Appendix A

### Appendix A.1. MRI Machine Models and Manufacturers

**Table A1:** MRI machine models and manufacturers.

Manufacturer	Model	Tesla
Philips Medical Systems	Achieva dStream	3
Philips Medical Systems	Ingenia Elition X	3
Philips Medical Systems	Ingenia Elition S	3
Philips Medical Systems	Ingenia	1.5 or 3
Siemens Healthineers	MAGNETOM Vida	3
Siemens Healthineers	MAGNETOM Aera	1.5
Siemens Healthineers	MAGNETOM Verio	3
Siemens Healthineers	MAGNETOM Avanto fit	1.5
Siemens Healthineers	MAGNETOM Avanto DOT	1.5
Siemens Healthineers	MAGNETOM Skyra	3
Siemens Healthineers	MAGNETOM Sola	1.5
GE Medical Systems	Discovery MR750	3

### Appendix A.2. Performance Evaluation Metrics

**Lesion-based Metrics**
Lesion-based Recall (Sensitivity):

This metric measures the model’s ability to detect all clinically relevant lesions. A higher recall indicates fewer missed lesions, which is crucial for comprehensive lesion detection. It can be calculated as

(A1) $${\mathrm{Recall}}_{\mathrm{Lesion}}=\frac{\mathrm{True}\;\mathrm{Positive}\;\mathrm{Lesions}\;{(\mathrm{TP}}_{l})}{\mathrm{True}\;\mathrm{Positive}\;\mathrm{Lesions}\;{(\mathrm{TP}}_{l})+\mathrm{False}\;\mathrm{Negative}\;\mathrm{Lesions}\;{(\mathrm{FN}}_{l})},$$

where ${\mathrm{TP}}_{l}$ represents the number of lesions detected by the fully automated AI/AI-assisted models that overlap with a lesion identified by the radiologist, and ${\mathrm{FN}}_{l}$ represents the number of lesions identified by the radiologist that do not overlap with any lesions from the AI/AI-assisted model.

Lesion-based Precision:

Precision evaluates the accuracy of the detected lesions. A high precision value ensures that most of the identified lesions are truly present, reducing false positives. It can be calculated as

(A2) $${\mathrm{Precision}}_{\mathrm{Lesion}}=\frac{\mathrm{True}\;\mathrm{Positive}\;\mathrm{Lesions}\;{(\mathrm{TP}}_{l})}{\mathrm{True}\;\mathrm{Positive}\;\mathrm{Lesions}\;{(\mathrm{TP}}_{l})+\mathrm{False}\;\mathrm{Positive}\;\mathrm{Lesions}\;{(\mathrm{FP}}_{l})},$$

where ${\mathrm{FP}}_{l}$ represents the number of lesions detected by the fully automated AI or AI-assisted models that do not overlap with any lesions identified by the radiologist.

**Voxel-based Metrics**
Dice Score:

To assess the spatial overlap of the segmented regions, the Dice score (DSC) was calculated [28]. The Dice Coefficient measures the overlap between the predicted and reference segmentations. It ranges from 0 to 1, where 1 indicates perfect agreement between the two segmentations, demonstrating accurate segmentation. The Dice score is calculated as

(A3) $$\mathrm{DSC}=\frac{2|A\cap B|}{\left|A|+|B\right|},$$

where |A| and |B| represent the total number of voxels in the predicted and reference segmentation masks, respectively. |A∩B| represents the count of voxels overlapping in both segmentations.

Hausdorff Distance:

The Hausdorff distance captures the largest boundary discrepancy between the predicted and reference segmentations. A lower value signifies a better match, highlighting areas with the greatest segmentation error [29]. The Hausdorff distance represents the greatest of all the distances from the set of boundary points in the predicted segmentation *A* to the closest set of boundary points in the reference segmentation *B*.

Hausdorff distance can be calculated as

(A4) $$HD\left(A,B\right)=\max\left(\underset{a\in A}{\sup}\underset{b\in B}{\inf}d\left(a,b\right),\underset{b\in B}{\sup}\underset{a\in A}{\inf}d\left(a,b\right)\right),$$

where d(a,b) represents the Euclidean distance between points a∈A and b∈B. sup represents the supremum (least upper bound) and inf represents the infimum (greatest lower bound).

In this formula, $\inf_{b\in B}d\left(a,b\right)$ is the shortest distance from a point a∈A to its closest point in *B*. Similarly, $\inf_{a\in A}d\left(b,a\right)$ is the shortest distance from a point b∈B to its closest point in *A*. The terms $\sup_{a\in A}\inf_{b\in B}d\left(a,b\right)$ and $\sup_{b\in B}\inf_{a\in A}d\left(b,a\right)$ represent the largest of these shortest distances for points in *A* and *B*, respectively.

A smaller HD value indicates better alignment, reflecting fewer outliers or significant discrepancies, while a larger HD value highlights areas of substantial error. This metric is particularly useful for identifying extreme misalignments.

Average Surface Distance:

To assess the average alignment of the boundaries, the Average Surface Distance (ASD) was calculated. This metric evaluates the average boundary alignment, providing insight into how closely the predicted and reference segmentations match, with smaller values signifying better alignment. The ASD is calculated as

(A5) $$\mathrm{ASD}=\frac{1}{\left|P|+|Q\right|}\left(\sum_{p\in P}d\left(p,Q\right)+\sum_{q\in Q}d\left(q,P\right)\right),$$

where *P* and *Q* are the sets of boundary points in the predicted segmentation and reference segmentation, respectively. The terms |P| and |Q| represent the number of boundary points in the predicted and reference segmentations. The distance d(p,Q) denotes the shortest Euclidean distance from a boundary point p∈P to the closest point in *Q*, and d(q,P) represents the shortest Euclidean distance from a boundary point q∈Q to the closest point in *P*.

Unlike the Hausdorff distance, which captures the worst-case error, the ASD focuses on the overall alignment quality, offering a more generalized view of segmentation performance. This metric is useful for evaluating the smoothness and precision of boundary matches between the predicted and reference segmentations.

Centroid Euclidean Distance:

To assess how effectively the centers of the segmentation masks are aligned, the Euclidean distance between the centroids of the estimated masks and reference masks was calculated as

(A6) $$C_{d}\left(p,q\right)=\sqrt{{\left(x_{2}-x_{1}\right)}^{2}+{\left(y_{2}-y_{1}\right)}^{2}+{\left(z_{2}-z_{1}\right)}^{2}},$$

where $x_{1},y_{1},z_{1}$ represent the coordinates of center point *p* in 3D space, and $x_{2},y_{2},z_{2}$ represent the coordinates of point *q* in 3D space. A smaller distance indicates better localization accuracy.

Voxel-based Recall and Precision:

To quantify the model’s ability to correctly classify all voxels that belong to the prostate or tumor, the recall was calculated, ensuring that relevant voxels are included in the segmentation. Additionally, voxel-based precision evaluates how accurately the model identifies positive voxels. Higher precision indicates fewer false positives within the segmentation. Voxel-based recall and precision are calculated as

(A7) $${\mathrm{Recall}}_{\mathrm{voxel}}=\frac{\mathrm{True}\;\mathrm{Positive}\;\mathrm{Voxels}\;\left(\mathrm{TP}\right)}{\mathrm{True}\;\mathrm{Positive}\;\mathrm{Voxels}\;\left(\mathrm{TP}\right)+\mathrm{False}\;\mathrm{Negative}\;\mathrm{Voxels}\;\left(\mathrm{FN}\right)},$$

(A8) $${\mathrm{Precision}}_{\mathrm{voxel}}=\frac{\mathrm{True}\;\mathrm{Positive}\;\mathrm{Voxels}\;\left(\mathrm{TP}\right)}{\mathrm{True}\;\mathrm{Positive}\;\mathrm{Voxels}\;\left(\mathrm{TP}\right)+\mathrm{False}\;\mathrm{Positive}\;\mathrm{Voxels}\;\left(\mathrm{FP}\right)},$$

where true positives (TPs) represent the proportion of voxels where the model correctly identifies a positive voxel, false negatives (FNs) represent the proportion of voxels where the model misses a positive voxel, and false positives (FPs) represent the proportion of voxels where the model incorrectly identifies a negative voxel.

**Volume-based Metrics**

To obtain a comprehensive understanding of the size of the annotated volumes, the volumes of the segmentations were calculated. Mathematically, it can be expressed as

(A9) $$V=N\times V_{\mathrm{size}},$$

where *V* represents the total volume of the segmentation, *N* represents the number of voxels in the annotated region, and $V_{\mathrm{size}}$ represents the volume of a single voxel.

The volume size of a single voxel can be expressed as

(A10) $$V_{\mathrm{size}}=d_{x}\times d_{y}\times d_{z},$$

where $d_{x}$, $d_{y}$, and $d_{z}$ represent the voxel size in mm in the *x*, *y*, and *z* directions, respectively.

Absolute Volume Difference:

This metric quantifies the absolute difference in volume between the predicted and reference segmentations. A smaller value indicates closer volumetric consistency. The Absolute Volume Difference can be expressed as

(A11) $$V_{\mathrm{diff}}=\left|V_{\mathrm{pred}}-V_{\mathrm{ref}}\right|,$$

where $V_{\mathrm{diff}}$ represents the difference in volume, $V_{\mathrm{ref}}$ represents the volume of the manual segmentation, and $V_{\mathrm{pred}}$ represents the volume of the AI tool segmentation or the AI-assisted segmentation.

Relative Volume Difference:

The Relative Volume Difference can be expressed as

(A12) $$V_{\mathrm{rel}}=\frac{V_{\mathrm{diff}}}{V_{\mathrm{ref}}},$$

which shows the Volume Difference as a percentage of the reference segmentation, highlighting proportional size accuracy.

### Appendix A.3. Subgroup Analysis of Lesion Segmentation Performance

The PI-RADS 3 group, containing only 5 lesions, was excluded from further analysis due to the small sample size. Among the metrics tested, only recall in the PI-RADS 5 group exhibited a normal distribution, with a *p*-value of 0.40. Consequently, the Mann–Whitney U test was used to evaluate statistical differences between the subgroups.

In the comparison of PI-RADS scores, statistically significant differences were observed only for precision and Volume Difference metrics. No significant differences were found in any metrics between lesions located in the peripheral zone (PZ) and the transition zone (TZ). Detailed results are provided in Table A2 and Table A3.

**Table A2:** Segmentation performance metrics for tumor comparison based on PI-RADS 4 and PI-RADS 5 scores, including mean, standard deviation, median, and *p*-values for statistical differences. * p<0.05.

Metric	PI-RADS 4			PI-RADS 5			*p*-Value
	Mean	STD	Median	Mean	STD	Median	
Dice Coefficient	0.54	0.16	0.57	0.53	0.17	0.55	1.00
Hausdorff Distance [mm]	9.46	6.51	7.24	9.88	5.67	7.88	1.00
Average Surface Distance [mm]	1.70	1.51	1.35	1.99	1.15	1.78	0.21
Recall_voxel_	0.55	0.21	0.53	0.44	0.20	0.46	0.20
Precision_voxel_	0.64	0.23	0.69	0.79	0.17	0.84	*
Euclidean Distance Centres [mm]	2.77	2.82	2.19	3.15	2.34	2.52	0.80
Absolute Volume Difference (cc)	1.28	4.71	0.32	2.17	6.00	1.01	*
Relative Volume Difference	0.80	1.61	0.41	0.47	0.32	0.44	1.00

**Table A3:** Segmentation performance metrics for tumor comparison by prostatic region (peripheral zone and transition zone), including mean, standard deviation, median, and *p*-values for statistical differences.

Metric	Peripheral Zone			Transition Zone			*p*-Value
	Mean	STD	Median	Mean	STD	Median	
Dice Coefficient	0.52	0.16	0.56	0.50	0.22	0.61	1.00
Hausdorff Distance [mm]	9.13	5.18	7.42	14.62	9.16	14.26	0.64
Average Surface Distance [mm]	1.73	0.92	1.66	3.19	2.63	1.68	1.00
Recall_voxel_	0.48	0.20	0.51	0.43	0.20	0.48	1.00
Precision_voxel_	0.72	0.21	0.76	0.76	0.26	0.82	1.00
Euclidean Distance Centres [mm]	2.88	2.12	2.52	5.43	5.25	2.89	1.00
Absolute Volume Difference (cc)	0.90	1.01	0.56	8.73	15.26	1.49	0.66
Relative Volume Difference	0.64	1.32	0.41	0.92	1.63	0.44	1.00
